# The EVIDEM framework and its usefulness for priority setting across a broad range of health interventions

**DOI:** 10.1186/1478-7547-9-8

**Published:** 2011-05-19

**Authors:** Sitaporn Youngkong, Noor Tromp, Dereck Chitama

**Affiliations:** 1 Nijmegen International Center for Health Systems Research and Education (NICHE), Department of Primary and Community Care, Radboud University Nijmegen Medical Centre, Nijmegen, The Netherlands; 2 Health Intervention and Technology Assessment Program (HITAP), Ministry of Public Health, Nonthaburi, Thailand; 3 School of Public Health and Social Sciences-Muhimbili, University of Health and Social Sciences, Tanzania

## Commentary

This commentary responds to the article by Goetghebeur et al [1], which applies the EVIDEM (Evidence and Value: Impact on DEcision-Making) framework to evaluate growth hormone therapy for Turner syndrome patients. The EVIDEM framework is developed to assist decision-makers in healthcare decisions, and encompasses a multi-criteria decision analysis (MCDA) matrix consisting of 15 quantifiable, and six qualitative components of decision. With this comprehensive set of criteria, relevant experts can assess the performance of health interventions, and results are input for informed and transparent healthcare decisions.

Goetghebeur et al [1] propose that the EVIDEM can be used to compare various interventions across disease areas in order to prioritize interventions. They suggest that the EVIDEM can analyze single interventions, and the performance of competing interventions can subsequently be compared in a performance matrix. While we value the qualities of the EVIDEM because of its scope and breadth, we have doubts on the results consistency of the EVIDEM to compare competing interventions, particularly when setting priorities across broad healthcare service areas (e.g. in designing the national health benefit package) for two main reasons.

First, the EVIDEM framework ignores the contextual nature of priority setting process by assuming a set of universal priority setting criteria [2]. In reality, the priority setting process is context specific and different sets of criteria lead priority setting of health interventions in different contexts. As the examples of studies in Nepal [3], Chile [4], and Ghana [5] show, the set of criteria identified for using in priority setting of health interventions were different between countries. Therefore, we suggest that the setting of prioritization criteria needs to be locally determined or verified, implying that the EVIDEM needs to be flexible to allow change/modification of the components to suit the local context.

Secondly, the EVIDEM is vulnerable to interventions ranking inconsistency where performance evaluation of a broad range of competing interventions is mandated. For example, the EVIDEM framework requires different expert panels to assess the performance of every single intervention separately. This may lead to inconsistency of the results as different expert panels may have different considerations across the broad range of interventions to be assessed. As shown in the EVIDEM Turner Syndrome case study [1], the panel of experts estimated growth hormone intervention to achieve 41% of maximum value. However, in the absence of established explicit weights of criteria, it is not certain that the same panel will be consistent in evaluating different interventions, or that another panel of experts comes up with the same or similar value.

These arguments raise the question whether the approach of EVIDEM is locally meaningful and consistent when priorities are set for a range of interventions. To address its limitations, we propose a stepwise process to identify criteria and their weights, and rank ordered interventions.

We suggest that, to set priorities of a range of interventions within a certain context, a locally-meaningful set of criteria and their relative importance (i.e. weights) should be elicited by consulting relevant stakeholders. As a next step, the identified set of criteria and weights are then used to consistently assess the performance of the broad range of interventions.

To illustrate the method, we describe a study in Thailand [6], which defined explicit criteria to prioritize health interventions for the national health benefit package. Our study was conducted in five main steps. First, in a group discussion among multi-stakeholders, the six most important prioritization criteria (and their levels) were identified i.e. type of intervention, target groups of intervention, severity of disease, number of beneficiaries, value for money, and budget impact. This step ensured that the criteria was verified for the Thai context. Second, based on those six criteria, we designed a discrete choice experiment (DCE) questionnaire, an approach that facilitates MDCA, and distributed this among 24 national health policymakers, 55 health professionals, and 163 general populations. Third, our DCE analyses resulted in odds ratios (OR) per criterion level (i.e. target group criterion contains three levels: elderly, adult and children). The OR indicated the relative importance of incremental changes in criterion levels (compared to a reference level), to select an intervention (Table 1). For example, policymakers are 5.73 times more likely to select health interventions that target the children than interventions targeting the elderly. In this way the criteria and their weights were the same for every health intervention, and ranking consistency was achieved. Fourth, from the DCE results, we calculated the interventions' probability of being selected, by combining the performance of interventions on each criterion and the importance of that criterion. The probability of being selected resulted in a rank ordering of health interventions. Fifth, the rank ordering was an important input in an elaborative process among policymakers. In the study, we presented the different rank orderings from those three perspectives of stakeholders to policymakers for more elaborative discussion. This included consideration of non-quantifiable criteria in reaching consensus on the final health interventions priority list for the national health benefit package.

**Table 1:** Relative importance (Odds ratios) of criteria by perspective

		Perspectives (Odds ratios)		
		
Criteria	Levels	Policy makers	Health professions	General population
Type of intervention	Prevention for non-communicable diseases			
	Prevention for communicable diseases	**	2.50*	1.56*
	Treatment for non-communicable diseases	**	1.22	1.13
	Treatment for communicable diseases	**	1.88	1.41
Target group of intervention	Elderly			
	Adult	3.71*	3.93*	2.40*
	Children	5.13*	2.92*	2.45*
Severity of disease	Not severe			
	Moderate severe	6.29*	4.24*	2.48*
	Severe	43.42*	6.00	2.06*
Number of beneficiaries	Few			
	Many	19.97*	8.64*	2.80*
Value for money	High cost but low effectiveness			
	High cost and high effectiveness	48.91*	23.27*	9.35*
	Low cost and low effectiveness	1.35*	2.28*	1.51*
	Low Cost but high effectiveness	31.60*	27.97*	12.96*
Budget impact	High budget impact			
	Low budget impact	9.91*	4.43*	4.25*
Log likelihood		-199.5608	-637.7022	-2301.6025
Pseudo R^2^		0.5065	0.3341	0.2055

*Significant variables (p < 0.05)

**Removed variable

Note: 1 The odds ratios were overestimated because of the small sample size of policy makers. However, there was no any relevance for the interpretation of the results.

2 The group of policy makers expressed higher preference on the high cost and highly effective interventions rather than the low cost with highly effective ones. The explanation of this is reported elsewhere.

The explicit weighing of criteria analyzed from DCE may improve the consistency of priority setting across contexts and over time, but does not solve the more fundamental problem that views of stakeholders, and therefore their expressed weights, may diverge. This is acknowledged by the 'Accountability for Reasonableness' (A4R) framework [7,8] which is based on the believe that any consensus on priority setting weights and subsequent results may be difficult to achieve because of these distinct perspectives of stakeholders. Instead of attempting to resolve the problem of diverse stakeholders' views, the A4R framework proposes to concentrate on a fair priority setting process. On this basis, when conditions of reasonableness, publicity, appeal and enforcement are satisfied, it would lead to decisions that are considered fair and acceptable to stakeholders. We agree with this point of view, and see much scope to integrate MCDA and A4R. The A4R framework has been criticized for not being more operational on the condition of 'reasonableness' [9] and the explicit definition and weighing of criteria we propose can be a response to that. This will foster the discussion among stakeholders in the priority setting process and render the process, if some consensus is reached on weights, also more consistent.

In summary, the framework of EVIDEM can be a useful tool to assess single intervention or to prioritize between only few interventions; however, in this paper, we place emphasis on the potential of DCE for consistently setting priorities between a range of interventions at once and its meaningfulness across different contexts.
