# The EVIDEM framework and its usefulness for priority setting across a broad range of health interventions

**DOI:** 10.1186/1478-7547-9-16

**Published:** 2011-10-26

**Authors:** Sitaporn Youngkong, Noor Tromp, Dereck Chitama

**Affiliations:** 1 Nijmegen International Center for Health Systems Research and Education (NICHE), Department of Primary and Community Care, Radboud University Nijmegen Medical Centre, Nijmegen, The Netherlands; 2 Health Intervention and Technology Assessment Program (HITAP), Ministry of Public Health, Nonthaburi, Thailand; 3 School of Public Health and Social Sciences-Muhimbili, University of Health and Social Sciences, Tanzania

## Correction

After the publication of this article [1], we became aware that two last sentences in the paragraph relied on original ideas following personal communication with a researcher, and should not have been presented here. Consequently, the reference number 9 which was cited for the removed issue should be taken from the article. The correct paragraph is provided below:

The explicit weighing of criteria analyzed from DCE may improve the consistency of priority setting across contexts and over time, but does not solve the more fundamental problem that views of stakeholders, and therefore their expressed weights, may diverge. This is acknowledged by the 'Accountability for Reasonableness' (A4R) framework [2,3] which is based on the believe that any consensus on priority setting weights and subsequent results may be difficult to achieve because of these distinct perspectives of stakeholders. Instead of attempting to resolve the problem of diverse stakeholders' views, the A4R framework proposes to concentrate on a fair priority setting process. On this basis, when conditions of reasonableness, publicity, appeal and enforcement are satisfied, it would lead to decisions that are considered fair and acceptable to stakeholders. In our view, exploring how stakeholders' divergent perspectives on the weighting of criteria can be met fairly, is an object for further research.

We regret any inconvenience that these corrections might have caused.
